# Mouse Ganglion-Cell Photoreceptors Are Driven by the Most Sensitive Rod Pathway and by Both Types of Cones

**DOI:** 10.1371/journal.pone.0066480

**Published:** 2013-06-07

**Authors:** Shijun Weng, Maureen E. Estevez, David M. Berson

**Affiliations:** 1 Department of Neuroscience, Brown University, Providence, Rhode Island, United States of America; 2 Institute of Neurobiology, Institutes of Brain Science and State Key Laboratory of Medical Neurobiology, Fudan University, Shanghai, P.R. China; Dalhousie University, Canada

## Abstract

Intrinsically photosensitive retinal ganglion cells (ipRGCs) are depolarized by light by two mechanisms: directly, through activation of their photopigment melanopsin; and indirectly through synaptic circuits driven by rods and cones. To learn more about the rod and cone circuits driving ipRGCs, we made multielectrode array (MEA) and patch-clamp recordings in wildtype and genetically modified mice. Rod-driven ON inputs to ipRGCs proved to be as sensitive as any reaching the conventional ganglion cells. These signals presumably pass in part through the primary rod pathway, involving rod bipolar cells and AII amacrine cells coupled to ON cone bipolar cells through gap junctions. Consistent with this interpretation, the sensitive rod ON input to ipRGCs was eliminated by pharmacological or genetic disruption of gap junctions, as previously reported for conventional ganglion cells. A presumptive cone input was also detectable as a brisk, synaptically mediated ON response that persisted after disruption of rod ON pathways. This was roughly three log units less sensitive than the rod input. Spectral analysis revealed that both types of cones, the M- and S-cones, contribute to this response and that both cone types drive ON responses. This contrasts with the blue-OFF, yellow-ON chromatic opponency reported in primate ipRGCs. The cone-mediated response was surprisingly persistent during steady illumination, echoing the tonic nature of both the rod input to ipRGCs and their intrinsic, melanopsin-based phototransduction. These synaptic inputs greatly expand the dynamic range and spectral bandpass of the non-image-forming visual functions for which ipRGCs provide the principal retinal input.

## Introduction

Intrinsically photosensitive retinal ganglion cells (ipRGCs) transduce light through their photopigment melanopsin and drive circadian and other non-image forming (NIF) visual responses [1], [2], [3], [4], [5], [6], [7], [8], [9], [10]. Light also affects ipRGCs through synaptic networks driven by rods and cones [11], [12], [13], [14], [15], [16], [17], [18], [19], [20]. Light-driven NIF responses persist when ipRGC phototransduction is silenced [3], [4], [5], [20], [21], but not when ipRGCs are killed [22], [23], [24]. Thus, ipRGCs link classical photoreceptors to NIF visual mechanisms.

Much remains unclear about which classical photoreceptors affect ipRGCs, and about the neural circuits that convey those influences. Primate ipRGCs receive both rod and cone input [11]. This has not been confirmed in rodents, the principle models for studies of the NIF system, although indirect evidence is consistent. Rodent ipRGCs receive light-driven synaptic inputs >100,000-fold more sensitive than melanopsin activation [12]. Both rod and cone bipolar cells contact ipRGCs [17], [18], [19] and NIF behaviors driven by ipRGCs reveal both rod and cone contributions [25], [26], [27]. Rod and cone signals are detectable in the suprachiasmatic nucleus [28], [29], which receives retinal input virtually exclusively from ipRGCs [30].

Synaptic responses of ipRGCs are driven predominantly by the ON channel [11], [12], [13], [14], but it is unclear which of the known ON pathways are involved: (1) the primary rod pathway, involving rod bipolar and AII amacrine cells; (2) the secondary rod pathway involving rod-cone coupling; (3) a direct input from rods to ON cone bipolar cells; or (4) the less sensitive cone pathway [31], [32], [33], [34], [35]. Cone inputs to primate ipRGCs exhibit spectral opponency (S-cone OFF; M+L-cone ON) [11]. In rodents, however, there are only two cone pigments (S and M) and in some retinal regions most cones express both pigments. Little is known about which of the two pigments influence ipRGCs and whether they mediate spectral opponency of the sort seen in primate ipRGCs.

Here, we assessed rod and cone inputs to mouse ipRGCs using extracellular recordings and genetic and pharmacological manipulation of circuitry. We document a very sensitive ON-channel input, presumably from the primary rod pathway, and a surprisingly sustained, non-opponent input from both S- and M-cones. These synaptic inputs expand the dynamic range and spectral bandpass of ipRGCs and, through them, of the NIF system.

## Materials and Methods

### Ethics statement

All experiments were strictly in accordance with National Institutes of Health guidelines for work with laboratory animals and were approved by the Institutional Animal Care and Use Committee at Brown University (permit #10100407).

### Animals

For multielectrode array recordings, we used adult C57BL6 mice (Charles River, Wilmington, MA, USA); or Cx36 knockout (KO) mice [36] and their WT (Cx36^+/+^) littermates. For whole-cell recordings, we used Opn4^cre/+^; Z/EG^+/−^ mice in which ipRGCs express green fluorescent protein, as described [37]. All mice were at least 8 weeks old and were housed for at least 7 days in a 12 hr light (∼200 lux): 12 hr dark (LD) cycle before the experiment. In order to control for diurnal fluctuations in retinal sensitivity [38], [39], [40], [41], [42], [43], all recordings were conducted 5–10 hours after light onset (Zeitgeber Time 5 to 10).

### Multielectrode array recordings

#### Retinal preparation

Mice were dark adapted overnight. All surgical procedures were performed under very dim red light. The mice were sacrificed by carbon dioxide asphyxiation and the eyes immediately enucleated. In bicarbonate-buffered Ames' medium (Sigma, St. Louis, MO, USA) equilibrated with 95% O_2_ and 5% CO_2_, the cornea was removed and the lens and vitreous extracted. The retina was dissected free from the eyecup and placed photoreceptor side down on a piece of filter membrane (Anodisc; Whatman, Piscataway, NJ, USA). Sometimes several very small radial cuts were made in the isolated retina before mounting to promote flattening. The mounted retina was transferred into the recording chamber of a multielectrode array (MEA-1060, Multi Channel System MCS GmbH, Reutlingen, Germany). The ganglion cell layer faced the array, which consisted of 59 recording electrodes, 10 µm or 30 µm in diameter, at a center-to-center spacing of 200 µm. A stainless steel ring was used to anchor the retina. The retina sat in the chamber for 1 hr before beginning recordings to permit stabilization of spike amplitudes. The retina was continuously superfused at 3–4 ml min^−1^ with oxygenated bicarbonate-buffered Ames' medium and maintained at 32°C with a temperature controller (TC-324B, Warner Instruments, Hamden, CT, USA). Under these recording conditions, ganglion-cell light responses were stable for at least 3 hours.

#### Recording and light stimulation

ipRGCs were identified by the sluggish, persistent light responses they exhibited in the presence of glutamatergic agents that interrupt transmission of signals from the outer retina to the ganglion cells (see below and Results). Amplified voltage data were digitized at 25 kHz (for recordings lasting a few seconds) or 10 kHz (for recordings of >1 min) using a PC-based A/D interface card and MC_Rack software (MCS). Signals were bandpass filtered (200 Hz to 3 kHz) and stored on a personal computer. Retinas were kept in complete darkness in the chamber, except when probed with test stimuli. Full-field light stimuli were generated with a tungsten-halogen microscopy illuminator (model EW-09741-50, Cole-Parmer Instruments, Vernon Hills, IL, USA) and delivered onto the retina by a fiber optic cable. Stimulus timing was controlled by a custom-made electromechanical shutter. Wavelength and intensity were adjusted by introducing narrow-band interference filters (10 nm bandwidth at half height) and neutral density filters (Newport Oriel Instruments, Stratford, CT, USA) into the light path. Light intensity was calibrated with a radiometer (S370, UDT Instruments, San Diego, CA, USA) and converted to the estimated time-averaged rate of photoisomerizations per rod per second (Rh^*^/rod/s), based on average rod density of 437,000 rods/mm^2^ [44], quantum efficiency of 0.67 [45], and fraction of incident light absorbed by the pigment of 0.2 [46]. Except when otherwise noted, we used a 500 nm narrowband stimulus (10 nm full width at half height) ranging in intensity from 2×10^−3^ to 2×10^4^ Rh^*^/rod/s. Stimuli were presented in a series that was monotonically ascending in intensity and identical for all experiments. Interstimulus intervals increased progressively within the series, ranging from 2 min between dim stimuli to 10 min between the brightest ones. This design permitted substantial recovery from the previous stimulus while reducing the total time needed to complete the series so as to avoid response rundown.

### Patch-clamp Recordings

Whole-cell voltage clamp recordings were made from GFP-positive ipRGCs in Opn4^cre/+^; Z/EG^+/−^ mice as previously described [47]. Retinas were dissected under dim red light, after making a small cautery mark on the dorsal cornea. A large relieving cut was made in the dorsal eyecup to permit later identification of the retinal location of recorded cells. Cells were recorded with glass pipettes (3–8 mΩ) containing (in mM): 120 K-gluconate, 5 NaCl, 4 KCl, or CsCl, 2 EGTA, 10 HEPES, 4 ATP-Mg, 7 phosphocreatine-Tris, 0.3 GTP-Tris, pH 7.3, 270–280 mOsm. Lucifer yellow was added to the recording pipette solution and passively diffused into the cell during the recording in order to allow for *post hoc* morphological identification of ipRGC subtype. Cells were voltage-clamped at −64 mV after correction for liquid junction potential. GFP-positive RGCs were targeted using mercury epifluorescence excitation, then maintained in darkness for 20–30 minutes before patching using infrared visual guidance. Based on our previous work [47], this targeting procedure resulted in a reduction in outer retinal sensitivity of approximately 2 log units. Thus, our whole-cell recordings were performed under conditions of extensive bleaching adaptation of rods so that synaptically mediated light responses in RGCs were presumably dominated by cone pathways. Diffuse full-field light steps for were provided by a beam from a xenon source passed through neutral density and narrow band filters as previously described [47].

### Chemicals

To isolate photoresponses attributable to phototransduction in ipRGCs, we used a cocktail designed to block glutamatergic signals from the outer retina. This included: 50 µM L (+) -2-amino-4-phosphonobutyrate (L-AP4, group III metabotropic glutamate receptor agonist), 40 µM 6,7-dinitroquinoxaline-2,3-dione (DNQX, AMPA/kainate receptor antagonist), and 30 µM D-2-amino-5-phosphonovalerate (D-AP5, NMDA receptor antagonist). The intrinsic melanopsin-based response was tested 15 to 20 min after the bath application of this cocktail. In some experiments, 100 µM meclofenamic acid (MFA) was added to the superfusate for at least 40 min to block retinal gap junctions. All pharmacological reagents were freshly prepared from aqueous stock solutions by dilution with Ames' medium. L-AP4, DNQX, and D-AP5 were obtained from Tocris (Ellisville, MO, USA) and MFA from Sigma-Aldrich (St. Louis, MO, USA).

### Data analysis

**Figure 1:**
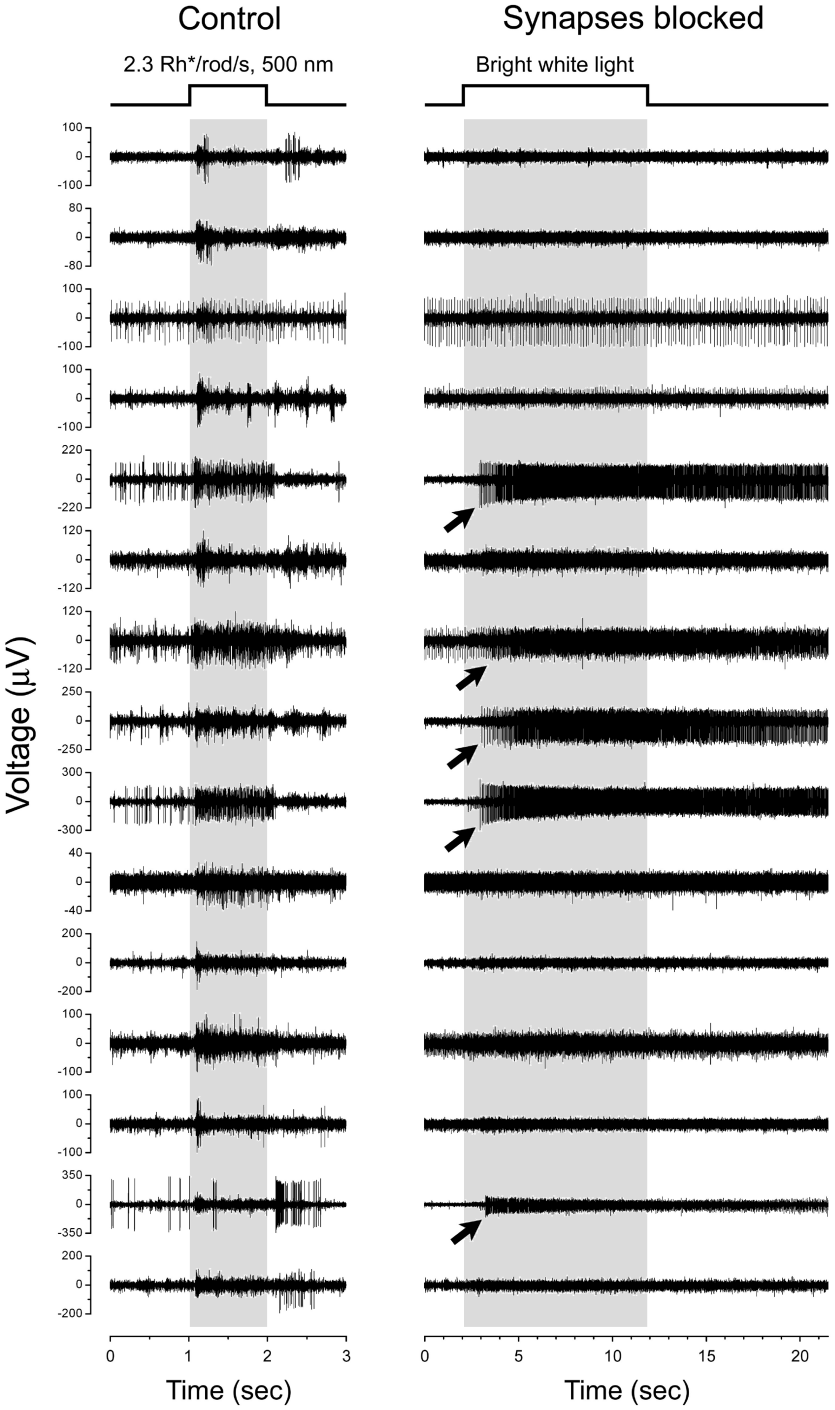
Pharmacological isolation of mouse ipRGCs . Representative raw voltage traces from a wildtype mouse retina (C57Bl6) recorded simultaneously from 15 electrodes of a multielectrode array. Most electrodes recorded spikes originating from more than one cell. Timing of full-field light stimulus is indicated by the step above the traces and by the time scales below the traces. ***Left column***: Spike responses recorded in control Ames' medium to a relatively dim scotopic flash (2.3 Rh^*^/rod/s; 500 nm; 1 second) that should excite rods but not cones or melanopsin[59]. Multiunit light responses are detectable on every channel, and exhibit various forms (ON, OFF or ON-OFF; transient or sustained). ***Right column***: Light-evoked spike activity recorded on the same electrode in the presence of drugs blocking rod and cone signaling to ganglion cells. To permit activation of melanopsin, the stimulus was longer and >10,000-fold brighter than that at left (∼2×10^5^ Rh^*^/rod/s sampled at 500 nm; 10 sec). This evoked responses on a minority of channels (arrows) and these exhibited the long onset latency and persistent post-stimulus discharge typical of melanopsin-mediated intrinsic responses (note the slower time scale).

Offline data analysis was performed using Offline Sorter software (Plexon Inc., Dallas, TX, USA). The spike detection threshold was set for each channel at 3-4 times the standard deviation of the voltage. The detected spike waveforms were subjected to cluster analysis using the first three principal components; the resulting clusters were manually corrected for clustering errors. The times of arrival of all spikes from each resulting cluster were then used to reconstruct single-unit spike trains. These were checked for evidence of the expected refractory period; any cluster in which >3% spikes had interspike intervals <1 ms was excluded from further analysis. Spike trains were further processed with OriginPro 7.0 (OriginLab Corp., Northampton, MA, USA) and Microsoft Excel (Microsoft Corporation, Redmond, WA, USA) to generate irradiance-response plots as described below. Typically, a few units in each retina had to be omitted from further analysis due to inconsistent light responses or difficulties in spike sorting. The amplitude of the light-evoked response was calculated by subtracting the maximal spontaneous firing rate (recorded during the 1 sec preceding light stimulation) from the maximal firing rate during the light stimulus. These maximal firing rates were calculated by a moving average using a 50 ms bin. Plots of evoked spike frequency against stimulus intensity were fit by the Michaelis-Menten equation:
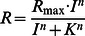



where ***R*** represents the measured response, ***R_max_*** represents the maximum response, ***I*** represents stimulus intensity, ***K*** represents the light intensity that induces a half-maximal response (0.5 ***R_max_***), and ***n*** is the Hill coefficient. Response thresholds were calculated from the Michaelis-Menten fit as the light intensity inducing 5% of the maximal light response. To group data across cells within a genotype, firing rates of each cell were first normalized to that cell's maximal response at any intensity. Raw data were also converted into. ABF files with MC DataTool software (MCS), and then viewed with Clampfit 10.0 (Axon Instruments, Union City, CA, USA) or processed with OriginPro 7.0 to generate voltage records such as shown in Fig. 1.

To assist in interpreting the spectral sensitivity of cone driven inputs to ipRGCs, we constructed an extremely simple model. We used a standard opsin template function [48] to describe the spectral sensitivity of the UV and M cone pigments, assuming λ_max_ of 360 and 510 nm respectively. We then modeled the irradiance-response functions of an imaginary cell whose output was simply a weighted linear sum of the outputs of these two pigment mechanisms; the free parameter was the ratio of input from the two pigments, and ranged from pure UV pigment input through mixed inputs to pure M pigment input.

## Results

### Identification of ipRGCs by pharmacological isolation

In dark-adapted retinas of wildtype mice, a brief light step in the scotopic range evoked spiking on nearly every channel (Fig. 1, left column). These multiunit responses were diverse. Some occurred at light onset, and these ON responses could be sustained or transient. Other channels exhibited OFF or mixed ON-OFF responses. All responses were brisk, beginning <100 ms after light onset or offset. Because these light stimuli were well below the threshold for melanopsin-mediated spiking, the responses must reflect the synaptically mediated influences of the classical photoreceptors of the outer retina. Consistent with this interpretation, blockade of glutamate receptor signaling eliminated all such light-evoked spiking (data not shown). Some of the cells recorded were ipRGCs. These were revealed under synaptic blockade by increasing the intensity and duration of the light stimuli (Fig. 1, right column). Under this condition, a minority of channels exhibited light responses characteristic of the intrinsic photoresponses of ipRGCs, including long onset latencies (>1 sec), sustained spiking during the stimulus, and pronounced post-stimulus discharges lasting >10 sec (Fig. 1, right column, arrows). We are confident that all such neurons are true ipRGCs and not conventional ganglion cells that are electrically coupled to ipRGCs. Though ipRGCs are coupled to amacrine cells through gap junctions, they apparently lack such coupling to ganglion cells [49]. Furthermore, neither genetic nor pharmacological disruption of gap junctions reduces the incidence of presumed ipRGCs, that is, cells responding to light under blockade of chemical synapses [41]. In a single preparation such activity was typically detectable on 5 to 15 electrodes from which we could reliably extract by spike sorting the single-unit spike trains of 3-10 ipRGCs. Tests for intrinsic photosensitivity were deferred until the end of the experiment because conducting them at the outset would have compromised rod- and cone-mediated responses through light adaptation and possibly through residual drug effects.

### Synaptic inputs to mouse ipRGCs include the most sensitive rod pathway

**Figure 2:**
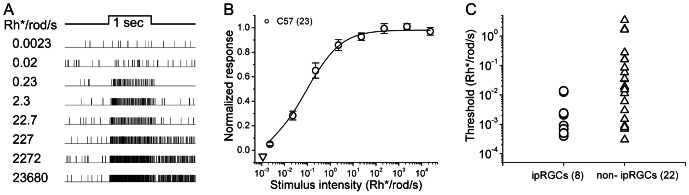
Input from the most sensitive rod pathway to ipRGCs. *****A*:**** Raster plots of synaptically driven spike responses of a single typical ipRGC in response to a series of flashes increasing in intensity (500 nm; 1 sec). Recordings were extracellular, obtained on a multielectrode array in a dark-adapted wildtype (C57Bl6) mouse retina. ***B***: Irradiance-response curve plotted in semi-log coordinates and based on data averaged from 23 wildtype mouse ipRGCs. For each cell, response amplitude at a given intensity was expressed as the firing rate during that stimulus normalized to the maximal light-evoked firing rate at any intensity. These values were averaged across all cells for each intensity and plotted, with error bars representing the standard error of the mean. The smooth curve represents the Michaelis-Menten fit to these points and the single triangle along the abscissa indicates the response threshold (5% of maximum, calculated from the fit). This irradiance-response profile closely resembles those of ON type RGCs receiving input from the primary rod pathway [50], [51]. ***C***: Distribution of the thresholds of the ON responses in ipRGCs (left; n = 8) in comparison with those of non-ipRGCs (right; n = 22) recorded in the same piece of retina. The thresholds of ipRGCs all located in the lower part of this plot, indicating they are among the most light-sensitive mouse RGCs.

In dark-adapted wildtype retinas, we probed the response of ipRGCs to a series of one-second light stimuli ascending in intensity and spanning the scotopic, mesopic, and photopic ranges (Fig. 2). Most ipRGCs showed brisk increases in firing to stimuli as dim as 0.02 Rh^*^/rod/s (Fig. 2A and B). Regardless of intensity, the responses were of the ON type. The maximal firing rate increased as a function of the stimulus intensity, and gradually saturated. Figure 2B illustrates the irradiance-response relationship for a pooled sample of 23 such ipRGCs. The data were well fit by the Michaelis-Menten equation as reported for synaptically driven light responses in other mouse RGCs [50], [51]. Response thresholds of ipRGCs, defined as the intensity producing 5% of the maximal response, averaged 1.1×10^−3^ Rh^*^/rod/s (n = 23). This is comparable to the thresholds of mouse ganglion cells driven by the most sensitive (primary) rod ON pathway relayed through rod bipolar cells and AII amacrine cells [50], [51]. Figure 2C plots the distribution of thresholds measured for the ON responses of 30 ON or ON-OFF RGCs, all recorded in a single piece of retina. Eight of these cells were ipRGCs (circles), and 22 were non-ipRGCs (triangles). The thresholds of the ipRGCs all fell into the lower half of the range exhibited by conventional RGCs. Thus ipRGCs appear to be among the most sensitive ganglion cells in the mouse retina, probably by virtue of an input from the primary rod pathway (see Discussion). Occasionally we encountered ipRGCs that could only be excited by strong light exceeding cone or even melanopsin threshold, but these were very rare (<< 1 cell per retina) and have been excluded from the analysis.

### Gap junctions are required for sensitive rod input to ipRGCs

To explore further the circuit basis of the sensitive rod input to ipRGCs, we exploited the connexin-36 knockout mouse (Cx36 KO) in which both the primary and secondary rod ON pathways are interrupted [51]. Signal transfer through the primary rod pathway is blocked in these mice because connexin 36 is an essential constituent of the gap junctions between AII amacrine cells and ON cone bipolar cells [51], [52]. The secondary rod pathway is also disrupted because connexin 36 is required for the gap junctional coupling between rods and cones [53], [54], [55], [56]. ON channel inputs to ganglion cells are much less sensitive in Cx36 KO mice than in wildtype mice [51], and the residual ON responses have been interpreted as arising from cone pathways, which are not dependent on gap junctional coupling.

**Figure 3:**
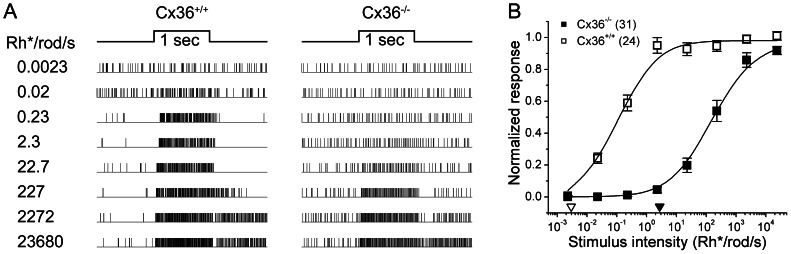
Light-evoked synaptic inputs to ipRGCs are less sensitive in Cx36 knockout mice. ***A***: Raster displays comparing responses of a single representative ipRGC in a Cx36 knockout mouse (right column) with those of an ipRGC in a wildtype littermate (left column). Responses of each cell are shown for a series of 500 nm flashes of increasing intensity. The dimmest stimulus evoking a clear response was approximately three log units higher in the Cx36 KO mouse (∼22.7 Rh^*^/rod/s) than it was in the wildtype control (0.02 Rh*/rod/s; see also the data for the C57Bl76 control mice; Fig. 2). ***B***: Group data comparing irradiance-response curves for ipRGCs recorded in the Cx36 knockout (black squares; n = 31) and wildtype animals (open squares; n = 24). Data come from 4 pairs of littermates consisting of one wildtype and one knockout animal. The triangles near the abscissa indicate the response thresholds (5% of maximum; open triangle, Cx36^+/+^; filled triangle, Cx36^−/−^), which are about 3 orders of magnitude higher in the knockouts than in the wildtype mice. This reduction in sensitivity presumably reflects the loss of input from the primary rod pathway.

Like other ganglion cells [51], ipRGCs were about three orders of magnitude less sensitive in Cx36 KO mice than in wildtype mice. The difference was just as large when we used wildtype littermate controls of the Cx36 mice instead of wildtype C57Bl6 mice as our basis for comparison (Fig. 3A and B). Thresholds in ipRGCs were roughly 3.0 Rh^*^/rod/s in Cx36 KO animals, as compared to about 1.1×10^−3^ Rh^*^/rod/s in wildtype mice (C57Bl6; Fig. 2), and about 3×10^-3^ Rh^*^/rod/s in wildtype Cx36^+/+^ littermates. The littermate control is important for excluding the possibility that the altered sensitivity was attributable to differences in genetic background between Cx36 KOs and C57Bl6 mice, rather than to the Cx36 knockout *per se*.

**Figure 4:**
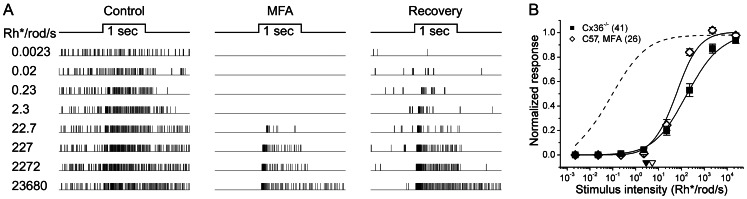
Pharmacological blockade of gap junctions mimics Cx36 knockout, reducing sensitivity of synaptic input to ipRGCs. ***A***: Raster plots of the response of a single ipRGC in a wildtype mouse (C57Bl6) to 500 nm light steps recorded before (left), during (middle) and after (right) bath application of MFA, a gap junction blocker. Stimuli increased in intensity by ∼1 log unit with each trial, shown in successive rows of the raster. MFA application abolished the light response to dim stimuli and elevated threshold by about 3 log units; spontaneous activity was also much reduced. Sensitivity largely recovered upon washout of the drug. ***B***: Group data comparing irradiance-response functions ipRGCs recorded in MFA-treated wildtype mouse retina (n = 26) with those recorded in Cx36 knockout mice (n = 41). The triangles near the abscissa indicate the response thresholds (open triangle, C57Bl6, MFA; filled triangle, Cx36^−/−^). The dashed curve, is reproduced from Fig. 2B, permits comparison with C57Bl6 mice under control conditions. The reduction in light sensitivity was comparable in these two manipulations, supporting the view that the effect is due in both cases to loss of synaptic circuits that are dependent upon gap junctions rather than to off-target genetic or drug effects.

To confirm that these knockout effects were directly attributable to the loss of Cx36 rather than to a developmental reorganization of retinal circuits secondary to the genetic manipulation, we studied in wildtype mice (C57Bl6) the effect of acute blockade of gap junctions on the synaptically mediated light responses of ipRGCs. Meclofenamic acid (MFA; 100 µM) is a potent non-specific gap junction blocker that has been shown to be highly effective in mammalian retina. It eliminates gap-junctionally mediated tracer coupling between retinal neurons [57] and greatly reduces the junctional conductance between AII amacrine cells and ON cone bipolar cells [58]. When this drug was added to the bath, ipRGCs behaved very much as they had in the Cx36 knockouts. They always failed to respond to relatively dim flashes in the scotopic range, but did exhibit brisk ON responses when stimuli were roughly three log units brighter than the threshold obtained under control conditions (Fig. 4A). These effects were partially reversible upon washout of the drug. Pooled irradiance-response data in the presence of MFA are plotted in Figure 4B (open diamonds) along with the very similar data obtained in Cx36 knockout mice (filled squares; a superset of the data plotted in Fig. 3B). We conclude that gap junctions are essential for the sensitive rod input to ipRGCs.

### Mixed cone input to ipRGCs

**Figure 5:**
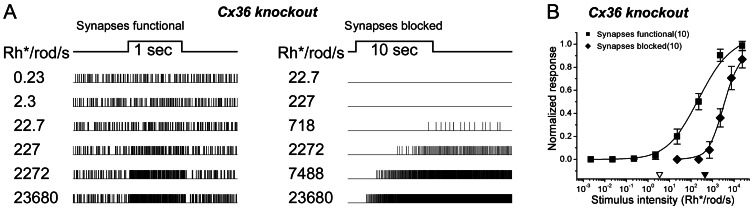
High-threshold light-evoked synaptic responses survive interruption of primary and secondary rod pathways by Cx36 knockout. ***A***: Raster plots of light responses in a representative ipRGC from a Cx36 KO mouse recorded first with synapses functional (left) and then with synapses blocked (right). In synaptic blockade, the light response exhibited the long latency and prominent poststimulus persistence of spiking characteristic of melanopsin-mediated responses. With synaptic input intact (left), responses were brisker and more sensitive than the melanopsin-driven response. Because both primary and secondary rod ON pathways were disrupted by the genetic deletion of Cx36, these brisk, synaptically mediated responses were presumably driven by cones. Note that stimulus intensities are not matched in the left and right columns of panel A. Also, stimulus duration was increased 10-fold under synaptic blockade (right) because a 1 sec stimulus was too short to evoke an intrinsic photoresponse at most intensities tested. ***B***: Group irradiance-response data of the sort illustrated in panel A for ten ipRGCs recorded from the same piece of Cx36 KO mouse retina. The triangles along the abscissa indicate the response thresholds (5% of maximum; open triangle, synapses functional; filled triangle, synapses blocked). The presumptive cone-driven response is at least 2 log units more sensitive than the melanopsin drive; the difference would have been greater if stimulus duration had not been increased under synaptic blockade to enhance the intrinsic response. Qualitatively similar results were obtained in a total of 8 retinas (data not shown).

Though ipRGCs in Cx36 KO mice lacked sensitive rod input, they still exhibited light-evoked synaptic input in addition to their intrinsic light response. One indication of this is the rapid onset and prompt termination of the light responses evoked near threshold (Fig. 3A, right panel), which differ markedly from intrinsic, melanopsin-mediated light responses (see Fig. 1, right panel). Similar brisk light responses were apparent when gap junctions were blocked pharmacologically (Fig. 4A, middle panel). The synaptic origin of the brisk responses was confirmed by their elimination by synaptic blockade (Fig. 5A, right panel), which left only the sluggish light responses typical of melanopsin-mediated intrinsic phototransduction (Fig. 4A, right panel). Thresholds for the intrinsic responses were about two log units higher than those of the synaptically mediated responses (Fig. 5B), although direct comparison is difficult because much longer stimuli were required to evoke the intrinsic responses (10 seconds for the data of Fig. 5).

**Figure 6:**
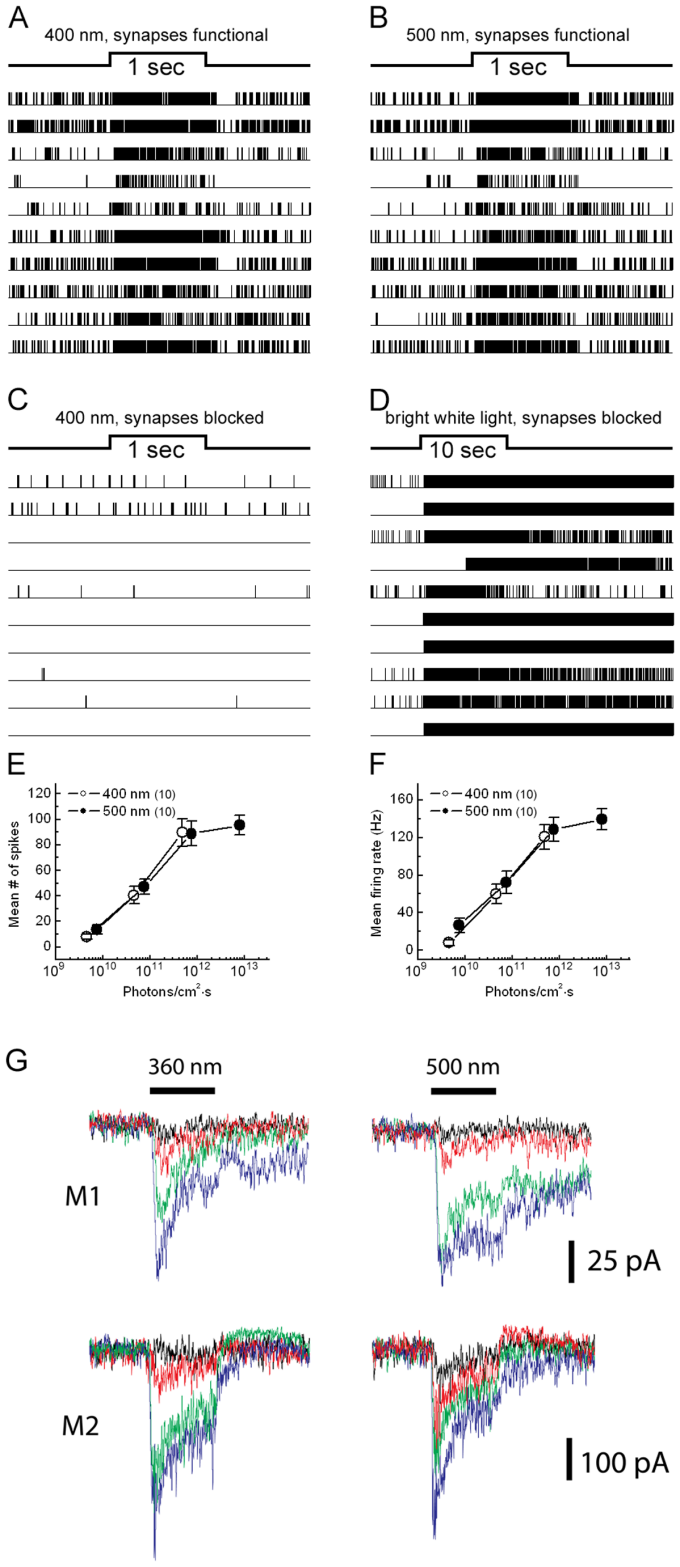
Evidence for ON channel input to ipRGCs from both M-cones and S-cones. **A**, **B**: Raster plots of responses to spectrally narrowband stimuli for ten ipRGCs recorded simultaneously in a Cx36 knockout mouse retina. These brisk ON responses were presumably exclusively cone-driven because Cx36 KO mice lack the primary and secondary rod ON pathways and because the flashes were subthreshold for activation by intrinsic melanopsin phototransduction. The response to a 400 nm stimulus (A) closely resembled that to a 500 nm stimulus (B), despite the fact that the former would be more effective in stimulating S-cones and the latter much more effective for M-cones. The two stimuli were approximately matched for photon flux density (400 nm: ∼5×10^11^ photons cm^−2^ s^−1^; 500 nm: ∼7×10^11^ photons cm^−2^ s^−1^). **C**: synaptic blockade eliminated these responses (in this case, the 400 nm response), confirming their origin in the outer retina. **D**: intrinsic, melanopsin-driven responses revealed in the same cells under synaptic blockade by increasing the intensity, spectral bandwidth and duration of the light. **E**, **F**: Group data summarizing the irradiance-response behavior at 400nm and 500nm for these cells (n  =  10), with response amplitudes measured either in terms of the number of evoked spikes (E) or the mean firing rate during the stimulus (F). The curves for the two wavelengths are nearly identical. This is inconsistent with a pure M-cone input, which would predict sensitivity at 500 nm ∼1 log unit greater than that at 400 nm. It is also inconsistent with a pure S-cone input, which would predict >4 log unit greater sensitivity at 400 nm than at 500 nm. We confirmed in a larger sample (25 ipRGCs from three Cx36 KO retinas) that 400nm and 500nm stimuli of matched irradiance evoked comparable responses. **G**: Whole-cell voltage clamp recordings from a representative example of an M1 cell (top panels) and an M2 cell (bottom panels) from the dorsal retina, where middle and long-wavelength cone opsins are expressed in distinct cone types. Responses are shown to a 1 s step of 360 nm (left) or 500 nm (right) light (horizontal black line). Different colored traces represent responses to different light intensities (in photons•cm^−2^•s^−1^) for the M1 cell: 3×10^11^−2×10^14^ (360 nm) and 2×10^12^−6×10^14^ (500 nm); and for the M2 cell: 7×10^10^−5×10^13^ (360 nm) and 1×10^11^−2×10^14^ (500 nm). Similar results were obtained in all M1 and M2 cells recorded, whether in the dorsal retina (six M2 cells and one M1 cell) or the ventral retina (four M2 cells and one M1 cell).

Which outer retinal photoreceptors mediate the brisk, relatively high-threshold ON response that survives the loss of gap junctional coupling but not the blockade of chemical synapses (Fig. 5B, squares)? Because both the primary and secondary rod ON pathways are blocked by the loss of electrical coupling, cones seem most likely to be responsible. However, a novel rod ON pathway has recently been identified that involves direct synaptic contacts between rods and a subset of ON cone bipolar cells [35], [59]. Because the pathway is carried entirely through chemical synapses, it should remain functional in Cx36 knockout mice and during pharmacological blockade of gap junctions. However, two lines of evidence argue against this additional rod pathway as the source of this influence. First, this pathway is reported to be as sensitive as the primary rod pathway [35], whereas the residual synaptic drive to ipRGCs in Cx36 knockouts is roughly 1000-fold less sensitive (Fig. 3B). Second, the spectral behavior of this residual synaptic response is inconsistent with a rod input. We probed ipRGCs from Cx36 KO mice with spectrally narrowband stimuli of 400 nm and 500 nm. Figure 6 A and B show the synaptically driven responses of ten such ipRGCs to narrowband stimuli of approximately matched irradiance, either 400 nm (Fig. 6A) or 500 nm (Fig 6B). Both wavelengths evoked brisk ON responses. These were clearly synaptically mediated because they were abolished by antagonists of chemical synaptic transmission (Fig. 6C). The irradiance-response functions obtained for the two wavelengths were virtually identical, using either of two response measures (Fig. 6E and F; see also [60]). This is inconsistent with a pure rod mediation of the response, which would have been expected to exhibit sensitivity nearly a full log unit higher at 500 nm than at 400 nm.

By contrast, the data of Fig. 6 are fully consistent with cone input, because the thresholds of Cx36-independent synaptic response are consistent with those of cones [51]. However, neither of the two murine cone photopigments in isolation can account for the observed spectral behavior: a pure M-opsin input would predict about a one log unit greater sensitivity at 500 nm than 400 nm sensitivity, whereas a pure S-opsin (UV-opsin) input would have resulted in more than 4 log units greater sensitivity at 400 nm than at 500 nm. However, the spectral behavior observed, both single-cell (data not shown) and group data (Fig. 6), can be reasonably well fit by a model in which there is convergent excitatory input from both cone opsins, of approximately matched strength (see Methods).

The implication that S and M cone opsins synergistically excite ipRGCs in Cx36 KO mice is consistent with an earlier report in mice [60] but seemingly at odds with the spectral antagonism reported in primate ipRGCs, in which S-cones drive OFF response, while mixed M- and L-cone inputs drive ON responses [11]. In Cx36 knockout mice, the 400 nm stimulus, which preferentially photoactivates S-opsin over M-opsin, invariably evoked pure ON responses in ipRGCs. This was true even for just-suprathreshold stimuli which would presumably have evoked negligible stimulation of M cones. We never observed obvious OFF responses with 400 nm stimuli at any intensity.

In mice, detecting cone opponency may be difficult because throughout much of the retina, the majority of cones express both short-wavelength and middle-wavelength cone opsins. In the dorsal retina, however, cones express only a single opsin; most cones express only middle wavelength cone opsin, while a minority of cones express only the short-wavelength opsin [61]. We therefore targeted several types of ipRGCs in the dorsal retina for whole-cell patch recordings under conditions that preserve robust cone inputs to ganglion cells [47]. As shown for a representative M1 and M2 cell in Fig. 6G, these cells invariably exhibited sustained ON responses to light at both short (360 nm) and long (500 nm) wavelengths. We have previously reported similar data on M4 cells [47]. Thus, even in a retinal region where S-cone and UV-cone opsins are expressed in distinct populations of cones, we were unable to detect any chromatic opponency. We also made voltage clamp recordings from several ipRGCs of the M2 type in the ventral retina and confirmed a lack of spectral opponency; all exhibited excitatory responses to UV as well as mid-visible wavelengths (n = 3; data not shown). A similar lack of opponency reported for a large sample of M2 cells of unspecified retinal locations [60]. M4-type ipRGCs are more strongly activated by UV cone opsin signals in the ventral than in the dorsal retina, but show no chromatic opponency in either retinal location [47]. To address the possibility of chromatic opponency in inhibitory input to ipRGCs, we voltage-clamped a small sample of ipRGCs at 0 mV to isolate inhibitory conductances. Though light-evoked post-synaptic currents varied among cells, none of these currents exhibited obvious spectrally opponent behavior (n  =  4; data not shown).

**Figure 7:**
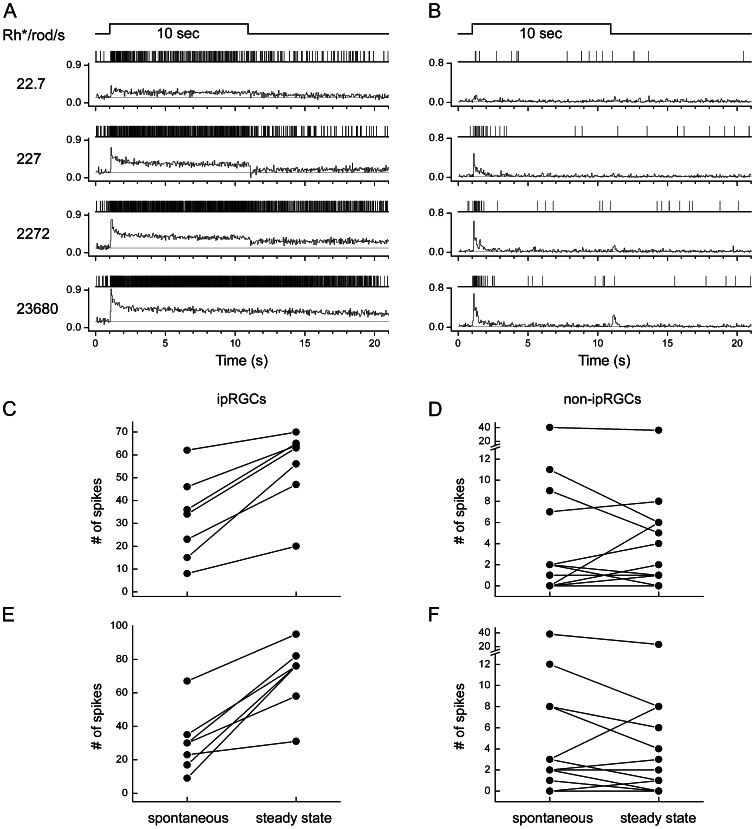
Surprisingly persistent cone-driven response in ipRGCs. Data are from a Cx36 knockout mouse retina, in which the primary and secondary rods paths are eliminated and ON channel synaptic input to ipRGCs comes mainly or exclusively from cones. **A**, **B**: Raster plots (above) and peri-stimulus time histograms (below) of responses of a single ipRGC (A) and of multiunit responses of conventional RGCs (B) to 10 sec light steps of various intensities (500 nm narrowband; n = 7 cells in A; n = 14 multiunit recording sites in B). Firing rates of each cell (or recording site) were first normalized to the maximal firing rate at any intensity, and then averaged over cells (or recording sites) to plot the histograms. Light intensities are listed at the left (in Rh*/rod/sec ). Light responses are always much more sustained in the ipRGCs than in the conventional ganglion cells, even at the two lower intensities, which are below the threshold for intrinsic, melanopsin-mediated phototransduction. Timing of light stimulus is indicated by the marker above the traces. **C–F**: Plots comparing of steady-state firing during the stimulus (right point) to the prestimulus spontaneous rate (left point) for individual ipRGCs (C, E) and for multiunit recordings of conventional RGCs (D, F). Spontaneous rate was assessed during the one-second interval preceding the stimulus, and steady state rate during the last second of the 10 sec stimulus. All data in C-F obtained simultaneously from a single Cx36 KO retina. Light intensities (in Rh*/rod/sec) are 22.7 in C and D; and 227 in E and F. Both of the intensities are below melanopsin threshold. Note that all ipRGCs showed elevated steady-state firing during prolonged stimulation, whereas nearly all conventional RGCs returned to baseline firing rates during the stimulus.

In a prior study in rat retina, we found that synaptically mediated ON responses were much more sustained in ipRGCs than in conventional ganglion cells [12]. We did not distinguish between rod- and cone-mediated responses in that study, though the sustained responses near threshold suggest that the rod system can support tonic responses in these cells. The Cx36 KO mice used in this study can shed new light on this issue because, as noted above, the synaptic drive to ipRGCs in these animals comes largely or exclusively from cones. In the knockouts, we find that synaptically mediated light responses are more sustained in ipRGCs than in conventional ganglion cells (Fig. 7). Thus it appears that both rod and cone circuits feeding ipRGCs may be specialized to provide tonic signals from the outer retina.

**Figure 8:**
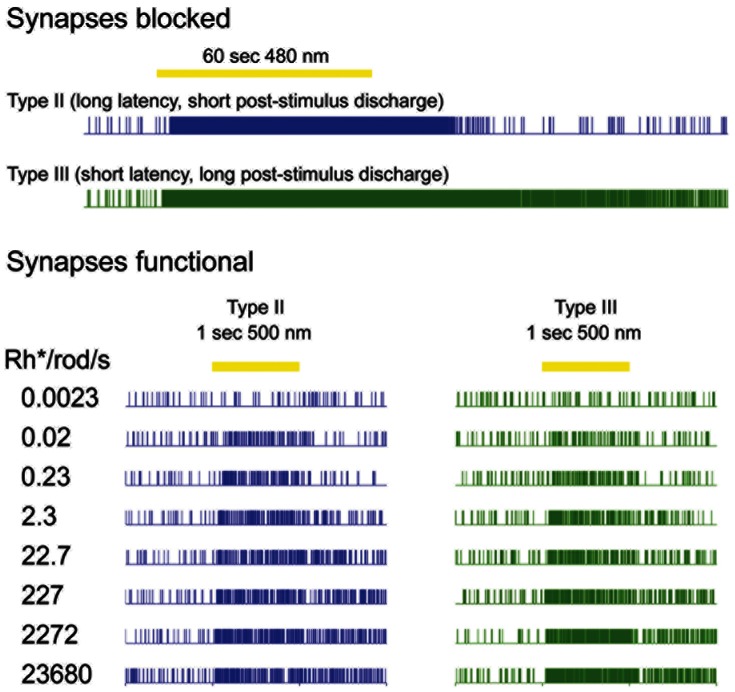
ipRGCs with distinct types of intrinsic light response exhibit similar synaptically driven photoresponses. Upper panel: raster plots of two representative ipRGCs with distinct kinetics of melanopsin-driven intrinsic photoresponse, apparently corresponding to two types defined by Tu et al., (2005) in adult mice. Type II cell (blue) has long onset latency and shorter post-stimulus discharge; Type III has short latency and prolonged afterdischarge. Stimulus was a 60 sec light step (yellow bar; 480 nm, ∼6×10^12^ photons/cm^2^·s). Recordings were performed under synaptic blockade. Lower panel: intensity-response series of the same two cells, but without synaptic blockade. The light stimuli were one second flashes 500 nm flashes ascending in intensity within the series. Both cells showed the same irradiance-response properties, with thresholds approximately 0.02 Rh*/rod/s.

We observed no difference in outer retinal drive to ipRGC subtypes distinguishable from the kinetics of their intrinsic light response (Fig. 8).

## Discussion

This study provides new insight into the influences of classical photoreceptors upon murine ipRGCs and the circuits that support them. They document a very sensitive ON-channel input from the primary rod pathway. They also reveal ON cone inputs that are surprisingly sustained, derived from both S- and M-cone opsins, and devoid of the chromatic opponency observed in primate ipRGCs. These synaptic influences complement the sustained intrinsic responses to bright light, allowing ipRGCs to report the presence of light continuously over an extremely large operating range.

### Rod inputs to ipRGCs

Earlier studies have implicated ipRGCs as a likely conduit for rod influence on the non-image-forming visual system. For example, direct contacts between rod bipolar cells and ipRGCs have been reported [17]. Rod signals have been detected in neurons of the suprachiasmatic nucleus [28]. As essentially the sole source of direct retinal input to the circadian master pacemaker [22], [30], [62], ipRGCs are the most likely source of these rod signals, although indirect paths cannot be excluded. In behavioral studies, circadian photoentrainment persists when cone and melanopsin signals are genetically silenced, leaving the rods as the sole functional photoreceptor [26], [27]. The present study provides the most direct functional evidence to date that rods contribute to the light responses of ipRGCs and are essential for responses at the low end of their dynamic range. We observed robust and sensitive rod input for all the ipRGCs we recorded. We almost certainly recorded from several, if not all, of the known subtypes of ipRGCs, although their subtype identity could not be determined in these experiments. Outer retinal drive did not differ among ipRGC subtypes distinguishable from the kinetics of their intrinsic light response (Fig. 8). These findings are superficially at odds with an earlier report that M1 cells receive markedly weaker synaptic drive than M2 cells [60]; however, that study entailed epifluorescence illumination of isolated retinas, which was assumed to strongly attenuate rod influence on ganglion cells, and thus cannot be directly compared with our study.

**Figure 9:**
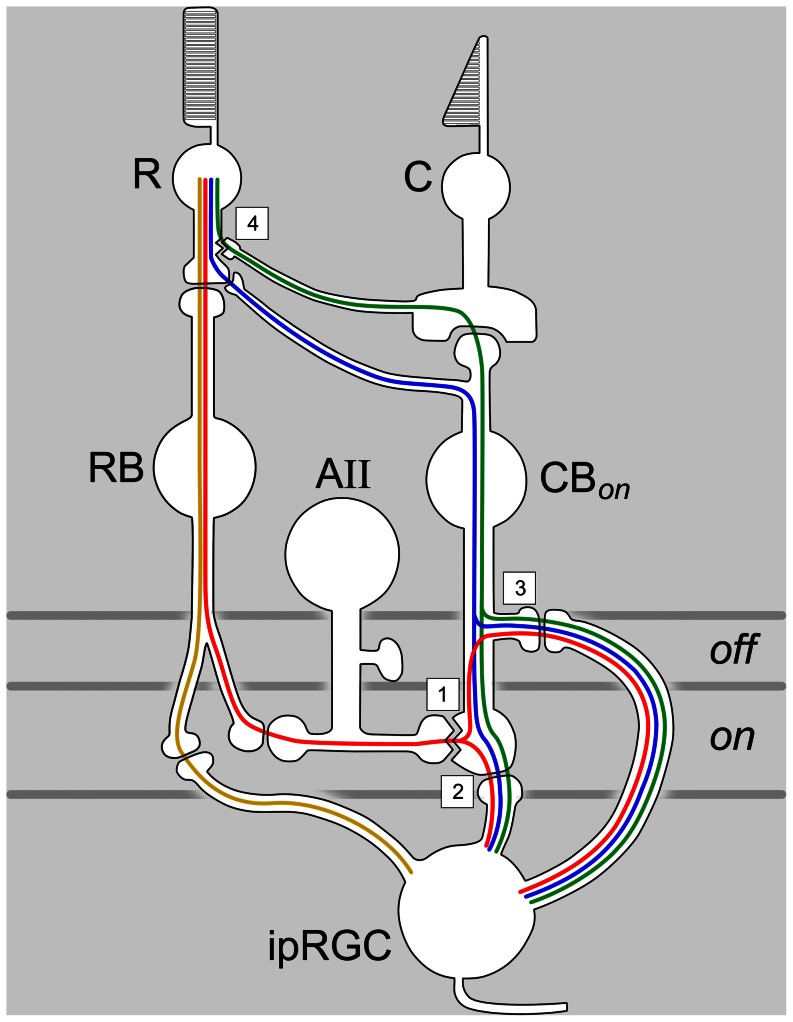
Schematic summary of pathways by which rod signals could reach ipRGCs. The ipRGC is a hybrid of known melanopsin-expressing ganglion cell types, some of which stratify exclusively in the uppermost OFF sublayer (site 3), others in the ON sublayer (e.g., site 2), and still others in both (see text). Jagged lines represent gap junctional contacts between AII amacrine cells and ON cone bipolar terminals (site 1) and between rods and cones (site 4). The primary rod pathway is shown in *red*, the secondary rod pathway in *green*, and a novel pathway from rods to ON cone bipolar cells in blue [35]. All of these pathways have been considered to relay to ganglion cells in the ON sublayer (site2), but they may also pass through ectopic ON cone bipolar terminals in the OFF sublayer to the dendrites of some ipRGCs (site 3; see text). A pathway directly linking rod bipolar cells to ipRGCs, proposed by Ostergaard et al. (2007) [17], is shown in *gold*. R: rod; C: cone; RB: rod bipolar cell; CBon: ON cone bipolar cell; AII: AII amacrine cell; ipRGC: intrinsically photosensitive retinal ganglion cell. Horizontal gray lines indicate the ON and OFF sublayers of the inner plexiform layer.

Figure 9 schematically summarizes four pathways that could transmit ON rod signals to ipRGCs. The high sensitivity of rod input to ipRGCs implicates the primary rod pathway (Fig. 9, *red pathway*) [31], [32], [33], [50], [51]. This conjecture is supported by the loss of sensitive rod input to ipRGCs in Cx36 knockout mice (Fig. 3), because Cx36 establishes a critical link in this circuit — coupling between AII cells and ON cone bipolar terminals (Fig. 9, *site 1*). The trajectory of the primary rod pathway may vary slightly among the various types of ipRGCs [11], [14], [30], [37], [63], [64], [65]. The M2, M4 and M5 ipRGC types deploy dendrites exclusively in the ON sublayer of the IPL, so signal transfer from rod bipolar cells presumably occurs conventionally, within that sublayer (Fig. 9, *sites 1 and 2*). Dendrites of the M1 ipRGC type, however, terminate only in the OFF sublayer where they receive ectopic synaptic contacts from certain ON cone bipolar cells [18], [19]. Primary rod signals may propagate in retrograde fashion in these ON cone bipolar axons, from their AII contacts in the ON sublayer (Fig. 9, *site 1*) to ectopic OFF-sublayer release sites (*site 3*). M1 cells may also receive ON cone bipolar synapses onto their proximal dendrites within the ON sublayer [15]. M3 cells are bistratified and could receive input by either route. Because our recordings were extracellular, we were unable to assess the morphological identity of the cells we recorded. However, our data offer no hints of major differences in outer retinal drive among different ipRGC types. The sensitivity of the synaptically driven inputs was invariably high, and there was no clear difference between ipRGCs with short or long poststimulus discharges, corresponding to Type II and Type III cells of Tu et al. (2005) (Fig. 8).

An alternate sensitive ON pathway involves direct contacts between rods and certain ON cone bipolar cells (Fig. 9, *blue pathway* [34], [35]). This pathway matches the sensitivity of rod bipolar cells [35], but cannot be the source of sensitive rod input to ipRGCs. In Cx36 knockout mice in which the primary rod pathway is silenced, the sensitive rod input to ipRGCs is lost too, despite the fact that this alternative pathway remains functional [35] [66]. Presumably, the cone bipolar cells that synapse upon ipRGCs lack direct rod input.

Ostergaard et al. (2007) propose yet another route for sensitive rod input to ipRGCs [17]. By light microscopy in rats, they observed direct rod bipolar synapses onto melanopsin-immunopositive dendrites in the ON sublayer of the IPL (Fig. 9, *gold pathway*). This observation is at odds with ultrastructural evidence that rod bipolars synapse only onto amacrine cells [67], [68], [69], [70]. Of course, ipRGCs account for only a small fraction of the ganglion-cell dendrites in the IPL, so such contacts may simply have been missed in these ultrastructural surveys. However, if the proposed contacts are present and functional, one would predict that a sensitive rod input to ipRGCs should survive disruption of gap junctions; our data are at odds with this prediction. Thus, sensitive rod input apparently reaches ipRGCs mainly, if not entirely, through the classical primary rod pathway, and not by way of direct contacts between rod bipolar cells and ipRGCs.

Our data are silent on the question of whether ipRGCs are influenced by the secondary rod pathway, involving rod-cone coupling (Fig. 9, *green pathway*) [46], [50], [51]. Some conventional ganglion cells with elevated rod thresholds have been reported to receive secondary but not primary rod input [50], [51]. However, in ipRGCs, the distribution of thresholds was unimodal and skewed toward the low thresholds typical of the primary pathway. Any secondary rod input to ipRGCs would have been masked by the more sensitive primary rod input and both would have been lost when we disrupted gap junctions (Fig. 9, *site 4*). Volgyi et al. (2004) selectively blocked the primary rod path by uncoupling AII and ON cone bipolar cells with S-nitroso-N-acetylpenicillamine (SNAP) and unmasked a secondary rod path input some ganglion cells [50]. SNAP did not affect sensitivity in the few ipRGCs we tested, but effective doses may not have reached the inner retina. Though the physiological evidence is thus inconclusive as to whether ipRGCs receive input from the secondary rod pathway, there is behavioral evidence suggesting that at least some ipRGCs do. Altimus et al. (2010) [26]compared photoentrainment in several strains of mice combining melanopsin knockout with various means of silencing cone pathways. Animals in which this was achieved by genetic ablation of cones (thus interrupting the secondary rod pathway) were unable to entrain under moderate light intensities whereas other “rod only” mice with intact (but photoinsensitive) cones could do so.

### Broadband cone inputs

When communication through gap junctions is disrupted, ipRGCs still exhibit brisk, synaptically mediated light responses, but with elevated thresholds (Figs. 3–6). These responses are ostensibly driven by cones because their sensitivity matches that of other cone-mediated responses in mouse retina [51], [71], [72]; neither the primary and secondary rod ON pathways can account for them because those pathways require electrical coupling (Fig. 9, *sites 1 and 4*). Though there are alternative rod ON paths that do not depend upon gap junctions [17], [35], these are known or presumed to be as sensitive as the primary rod path (see above). Finally, the spectral behavior of the presumptive cone-based responses is inconsistent with mediation by rods alone, but can easily be explained by a mixed excitatory input from M- and S-cone opsins (Fig. 6).

This inferred cone influence parallels independent electrophysiological, anatomical and behavioral observations. Presumptive synaptic contacts between certain ON cone bipolar cells and melanopsin-immunopositive dendrites have been observed [16], [18], [19], [64]. Functional studies of the synaptic drive to primate ipRGCs strongly implicate contributions from cone circuits [11]. Cone signals have been inferred to reach the rodent suprachiasmatic nucleus (SCN) [25], [27], [28], [29]; but see [26]; see also [73]. These are presumably transmitted through ipRGCs, which provide virtually the only source of direct retinal input to the SCN [22], [23], [24], [30], [62], [74], [75].

The cone drive to ipRGCs appears relatively sustained (Fig. 7; [12], [60], [76]). This is consonant with the ability of ipRGCs to encode irradiance stably over a wide dynamic range [11], [14], [75], [77]. It seems inconsistent, though, with behavioral evidence that cone influences on NIF systems are transient and can persist only if triggered by flicker [25], [27], suggesting pronounced light adaptation in this channel. It may be that the light stimuli we used were too brief to reveal this adaptation.

At intermediate photopic levels, it is possible that sustained subthreshold depolarization by intrinsic melanopsin phototransduction sums with weak sustained cone drive to support maintained responses to prolonged illumination. However, the brisk shutoff kinetics of these tonic responses and their dependence on synaptic transmission implicate at least a weak sustained cone drive. Furthermore, support from melanopsin is apparently not essential for sustained, cone-driven spiking; when intrinsic photosensitivity is silenced by genetic deletion of melanopsin and rods are saturated or heavily bleached, sustained light responses are still detectable in identified or presumed ipRGCs ([60], [76]). In these melanopsin knockout mice, cells with very tonic ON responses to photopic stimuli were just as common as in wildtype mice (unpublished observations).

Cones themselves support photoresponses lasting tens of minutes [78], [79]. Our findings suggest that high-pass temporal filtering is much stronger in synaptic circuits feeding conventional RGCs than in those reaching ipRGCs. This difference may be attributable to the specialized bipolar contacts onto ipRGCs [18], [19], though specializations in outer retinal synapses or in the electrical behavior of the ON cone bipolar cell types contacting ipRGCs may also contribute.

The mouse ipRGCs we studied lacked the S-cone-OFF, M/L-cone-ON chromatic opponency documented in primate ipRGCs [11]. Instead, light steps in invariably evoked excitatory ON responses in ipRGCs whether they were in the near ultraviolet or in the middle and long-wavelength end of the visible spectrum, in agreement with the earlier findings [47], [60]. Weak OFF responses can sometimes be recorded in rodent ipRGCs, especially when the ON pathway is blocked [12], but it is unknown whether rhodopsin, or either or both cone opsins drives this weak OFF channel input. This absence of spectral opponency is typical among ganglion cells in mice and other rodents, although some exhibit single-cone or opponent inputs [80], [81], [82], [83]. Most of our multielectrode array data were obtained in the central retina, where many cones express both cone opsins [61], [84], [85]. However, targeted whole cell recordings of M1 and M2 cells (Fig. 6G; and M. Estevez, unpublished observations) and M4 cells [47] in both dorsal and ventral retina confirmed the absence of chromatic opponency. The relatively strong input from UV cone opsin in the dorsal retina is unexpected given its low abundance there. This may hint at specialized circuits linking S-cone-selective ON bipolar cells to ipRGCs that may boost this opsin's influence. In any case, our data suggest that all four opsin photopigments in the retina drive ON responses in murine ipRGCs. Cone signals operate at irradiances between rod saturation and melanopsin threshold, and the S-cone inputs enhance the sensitivity to ultraviolet and blue light at such intensities. Why cone inputs are chromatically opponent in primates but not in mice remains mysterious. Comparative studies may reveal whether this discrepancy is linked to distinctions between dichromacy and trichromacy, or between nocturnal and diurnal lifestyles.
